# Subcellular Localization and Vesicular Structures of Anthocyanin Pigmentation by Fluorescence Imaging of Black Rice (*Oryza sativa* L.) Stigma Protoplast

**DOI:** 10.3390/plants10040685

**Published:** 2021-04-02

**Authors:** Enerand Mackon, Yafei Ma, Guibeline Charlie Jeazet Dongho Epse Mackon, Qiufeng Li, Qiong Zhou, Piqing Liu

**Affiliations:** State Key Laboratory of Conservation and Utilization of Subtropical Agro-Bioresources, College of Agriculture, Guangxi University, Nanning 530005, China; breedermackon@st.gxu.edu.cn (E.M.); mayafei@st.gxu.edu.cn (Y.M.); msmackon@st.gxu.edu.cn (G.C.J.D.E.M.); 1717303003@st.gxu.edu.cn (Q.L.); 19890006@gxu.edu.cn (Q.Z.)

**Keywords:** anthocyanin, stigma, protoplast, anthocyanin vacuolar inclusion, autofluorescence, confocal microscopy, subcellular localization

## Abstract

Anthocyanins belong to the group of flavonoid compounds broadly distributed in plant species responsible for attractive colors. In black rice (*Oryza sativa* L.), they are present in the stems, leaves, stigmas, and caryopsis. However, there is still no scientific evidence supporting the existence of compartmentalization and trafficking of anthocyanin inside the cells. In the current study, we took advantage of autofluorescence with anthocyanin’s unique excitation/emission properties to elucidate the subcellular localization of anthocyanin and report on the in planta characterization of anthocyanin prevacuolar vesicles (APV) and anthocyanic vacuolar inclusion (AVI) structure. Protoplasts were isolated from the stigma of black and brown rice and imaging using a confocal microscope. Our result showed the fluorescence displaying magenta color in purple stigma and no fluorescence in white stigma when excitation was provided by a helium–neon 552 nm and emission long pass 610–670 nm laser. The fluorescence was distributed throughout the cell, mainly in the central vacuole. Fluorescent images revealed two pools of anthocyanin inside the cells. The diffuse pools were largely found inside the vacuole lumen, while the body structures could be observed mostly inside the cytoplasm (APV) and slightly inside the vacuole (AVI) with different shapes, sizes, and color intensity. Based on their sizes, AVI could be grouped into small (Ф < 0.5 um), middle (Ф between 0.5 and 1 um), and large size (Ф > 1 um). Together, these results provided evidence about the sequestration and trafficking of anthocyanin from the cytoplasm to the central vacuole and the existence of different transport mechanisms of anthocyanin. Our results suggest that stigma cells are an excellent system for in vivo studying of anthocyanin in rice and provide a good foundation for understanding anthocyanin metabolism in plants, sequestration, and trafficking in black rice.

## 1. Introduction

Anthocyanin is a subcategory of flavonoid compounds found in plants providing numerous functions in Human bodies and plants themselves. Some scientific studies, such as animal models and human clinical trials, revealed that anthocyanins have potential antioxidant and antimicrobial properties [1], they are effective in supporting glucose homeostasis in the treatment of diabetes [2], cardiovascular diseases [3,4], and can prevent tumor and cancer [5,6,7]. They also possess anti-carcinogenic and anti-inflammation properties [8], improve visual and neurological health, and offer protection against various non-communicable diseases [9]. In plants, they act as a bio-protectant molecule against reactive oxygen species (ROS) formed during respiration and photosynthesis activities [10]. Moreover, under biotic and abiotic stress exposure, anthocyanin sustains plant growth and development as it can indirectly protect leaves from damage and maintain normal vital activity [11,12,13]. In the food industry, anthocyanins are often used as alternatives for food colorants [14]. Their color change with pH from red to blue in acidic and alkaline environments, respectively. These properties have raised the research interest in agriculture, health science, and commerce significantly.

Anthocyanins are found in various parts of the plant, such as flowers, leaves, stems, shoots, grains, and are responsible for a wide range of attractive colors like black, purple, pink, reddish, and orange [15]. Although the anthocyanin biosynthesis mechanism has been extensively studied and significant progress has been achieved [16], its storage mechanism and subcellular structure remain unclear. Lately, anthocyanin storage mechanism has gained more attention from different research programs. Numerous studies revealed that anthocyanins are synthesized on the surface of plant endoplasmic reticulum (ER) by a multi-enzymes complex [16,17,18]. Once produced, the anthocyanins are transported from the cytoplasm and store in the central vacuole via vacuolar sequestration in high concentration, which gives the intensely colored plant tissues [17,19]. These structures were first called anthocyanoplast because they were thought to be biosynthesis sites [20]; Further characterization described it as an evenly colored solution, vesicle-like bodies, and dense, compact bodies of either regular or irregular shape in cells termed anthocyanic vacuolar inclusion (AVI) [21,22].

Concerning the anthocyanins’ fate after synthesis, several studies reported different forms of anthocyanin either inside the cytoplasm or vacuole observed mostly with the light microscope in various species of angiosperms. In lisianthus, anthocyanins are stored in AVIs. These structures may have several forms, such as vesicle-like forms, irregular forms, and rod-like forms [23]. In grapevine, anthocyanins are accumulated in three types of vesicles localized outside and inside the vacuole called, respectively anthocyanic prevacuolar vesicles (APVs) and AVIs in part [22]. In *Arabidopsis*, AVIs and APVs were also identified in cells accumulating high anthocyanin quantity [24]. In sweet potato, the vesicles-like AVIs have been described as a fusion of a large number of small vesicles, which increase gradually in size [20]. Some studies reported that it might contain some protein components useful for binding specific anthocyanins [25]. For instance, in grapevine, AVI is rich in acylated anthocyanin [22,26], while in lisianthus, di-glucosidic anthocyanin has been reported [21] and cyanidin-3-glucoside (C3G) and derivatives in *Arabidopsis* [27].

A recent study revealed that AVI is formed by the micro-autophagy of cytoplasmic anthocyanin aggregates. Thus, many of these body structures found in the cytoplasm could correspond to either early or intermediate in AVI formation. This autophagic body may further move inside the vacuole through the vacuolar membrane’s evagination with the portion of the tonoplast surrounding this structure, which could eventually be free in the vacuolar lumen [28]. AVIs have potential commercial value as densely packed bodies of stabilized anthocyanins used as food additives. Therefore, further research on anthocyanin and AVI warrant more attention.

Black rice contains more than eighteen (18) types of anthocyanins, although only four are most frequently reported, namely C3G, peonidin-3-glucoside (P3G), cyanidin-3-rutinoside (C3R), and cyanidin-3-galactoside (C3Ga) [29]. These anthocyanins are present in different rice parts, such as leaves, grain, stigma, and stem, at the different developmental stages and can be easily recognized by a light-to intense purple, deep purple, and black coloration. The anthocyanin biosynthesis and storage in rice are complex processes in which several structural and regulatory genes are involved. In recent years, significant progress has been achieved in the molecular and genetic mechanism of anthocyanins, and their synthesis is now of great interest to the researchers and scientific community. However, limited studies on deciphering anthocyanin storage and subcellular activities are available.

This study highlights the cellular and subcellular localization of anthocyanin and reports in planta characterization of AVI structure in rice (*O. sativa*) using confocal and bright-field microscopy. In fact, like chlorophyll and many other biological compounds exhibiting autofluorescence with unique excitation/emission properties [30], anthocyanin can autofluorescent with in vitro excitation and emission peaks in UV [31,32], thus can be visualized based on their fluorescence properties in vitro and in vivo. This autofluorescence property of anthocyanin can offer a great opportunity for developing new tools used in the investigation of the dynamics of several metabolites inside the cells without any interference with exogenous markers and fluorescent dyes [33]. Many research studies reported using anthocyanin autofluorescence to form both simple intensity-based images and multidimensional quantitative maps of their fluorescence lifetime using fluorescence lifetime imaging microscope (Flim) [33,34,35].

Previous research showed that vacuolar anthocyanin could statically quench other fluorescent molecules in vivo [36]. Taking advantage of deep purple to purple color generated by anthocyanin’s autofluorescence, stigma protoplast under confocal microscope clearly showed AVI in rice cells. Our result indicated the presence of vesicular structures termed AVIs inside the vacuole and APV inside the cytoplasm. This result suggested that stigma cells are an excellent system for in vivo studying anthocyanin in rice. This result is the first reporting cellular and subcellular localization of anthocyanin and AVI in rice that may serve as the baseline study for anthocyanin accumulation and trafficking in black rice.

## 2. Results

### 2.1. Investigation of the Anthocyanin Pigmentation in Black Rice Tissues

**Figure 1:**
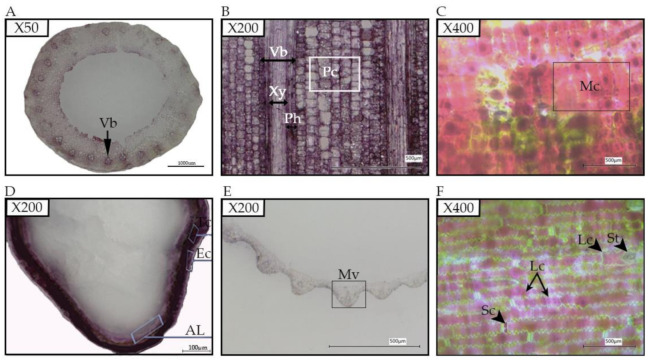
Anthocyanin pigmentation in different tissues of black rice; (**A**), a cross-section of stem showing vascular bundle (Vb) containing the anthocyanin; (**B**), leaf sheath longitudinal section showing a high amount of anthocyanin in parenchyma cells (Pc), xylem (Xy) and phloem (Ph); (**C**), the longitudinal section of ligule showing mesophyll cell (Mc) filled with the anthocyanin; (**D**), a cross-section of the caryopsis showing the aleurone layer (AL), the epidermal cells (Ec), and the transverse cells (Tc) colored by the anthocyanin; (**E**), a cross-section of a leaf blade with the midvein (Mv) containing the anthocyanin; (**F**), the longitudinal section of a leaf showing long cells (Lc) and silica cells (Sc) filled with anthocyanin, and stomate (st).

In black rice, anthocyanin is found at the different developmental stages in various tissues and organs, such as caryopsis, leaves blade, leaves sheath, internodes, stems ligules, apiculus, and stigmas and can be visualized with naked eyes or under a microscope. While in some black rice, only some tissues and organs are pigmented, whereas, in other cultivars, all parts are fully and intensely colored. Most of the black rice cultivars do not display pigmentation phenotype until plants are 3–4 weeks old in normal condition. In this study, our black rice 18BLN6321 exhibits pigmentation in all parts at the flowering stage and filling stage (Figure 1). The purple to dark purple color that remained in the epidermal cells of the sections provides a good marker to recognize the anthocyanin-containing cells. These results indicated that anthocyanin might be sequestrated inside the cells that could be identified after the section (Figure 1C,D,F). Anthocyanins are not only found in the epidermal layer but have been identified in the walls of conducting vessels (Figure 1A,B,E).

### 2.2. Anthocyanin Quantification in Rice Stigma and Caryopsis

**Figure 2:**
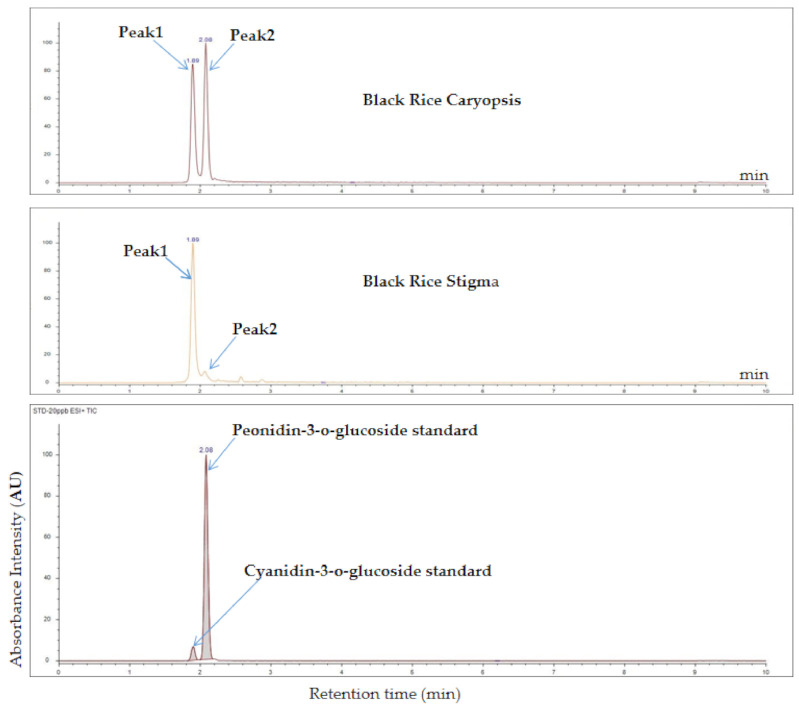
HPLC analysis of anthocyanins in the purple stigma and black caryopsis. Peak1 and peak2 represent cyanidin-3-glucoside (C3G) and peonidin-3-glucoside (P3G), respectively.

We performed high-performance liquid chromatography (HPLC) to analyze the two major rice anthocyanins (C3G and P3G) and one minor anthocyanin (petunidin-3-O-glucoside, Pt3G) in purple stigma (Figure 2) and black caryopsis (35 days after flowering). Our results showed that Pt3G was not detected, while C3G and P3G were detected in both black caryopsis and purple stigma. The amount of C3G in stigma was 16,929.14 ± 35 µg · g ^−1^ FW, which is about 20 times higher than that in the mature black caryopsis, whereas the amount of P3G was the same in both black caryopsis and purple stigma (35 µg· g ^−1^). We did not detect anthocyanin in white caryopsis (Table 1).

### 2.3. Black Stigma Revealed the Autofluorescence of Anthocyanin in Rice

**Figure 3:**
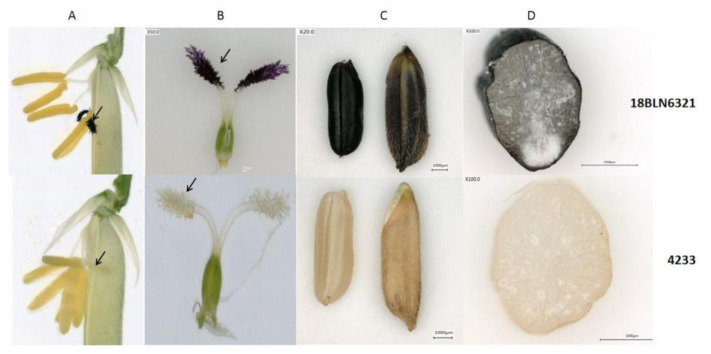
Characteristics of the two cultivars; the first row indicates a black rice cultivar 18BLN6321 and the second row indicates a brown rice cultivar 4233; (**A**), the flower of black and brown cultivar, the arrow indicates the stigma; (**B**), the pistil of both cultivar, the arrow indicates stigma; (**C**), black and white caryopsis; (**D**), a cross-section of black and white caryopsis.

Purple stigma is an important trait in black rice because black rice usually has colored stigma, although the intensity varies. Black rice cultivar 18BLN6321 used in this study showed a deep purple color of stigma, black grain, and caryopsis, while brown rice 4233 had a white stigma and brown caryopsis (Figure 3). Because of its opacity and deep color at the flowering stage, microscopic observation of fresh stigma does not allow the visualization of cells. Meanwhile, this was possible for other parts, including peel fresh leaf sheath, leaf blade, ligule, and stem. Here, the microscopic observation at a total magnification of 400× showed anthocyanin coloration diffused throughout the mesophyll cells (Figure 1C, distributed in long cells (Figure 1F). Another observation showed anthocyanin through the plants’ vascular tissues (xylem and phloem), cell wall, and parenchyma cells (Figure 1B). However, we could not isolate single cells from leaves sheath and rice brans. At the flowering stage, stigma is one of the smooth parts of rice with anthocyanin. This made it possible and easy for the isolation of protoplasts to be used for investigating cellular and subcellular localization of anthocyanin.

**Figure 4:**
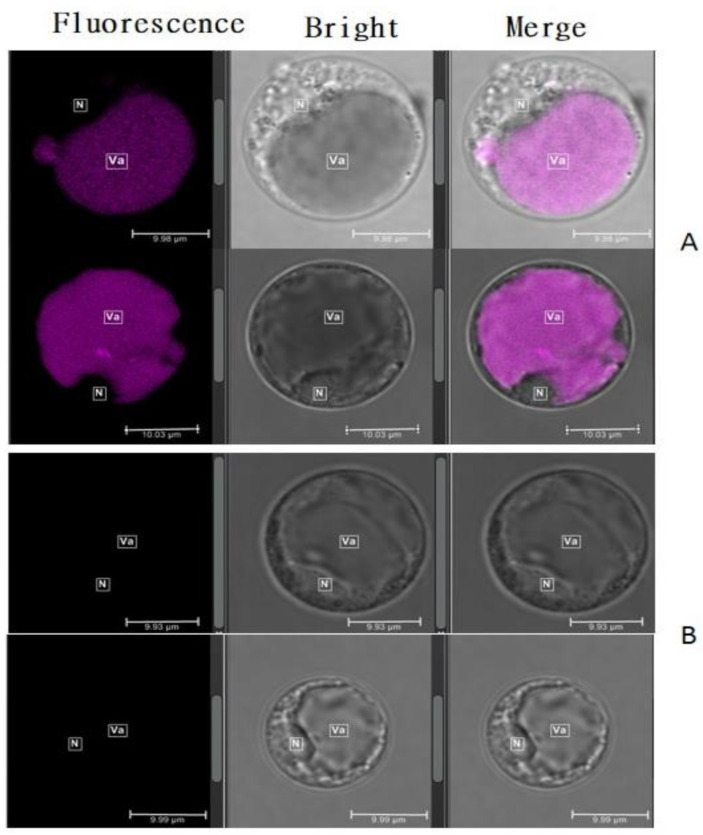
Confocal microscopy of unstained protoplast cells; (**A**), black rice 18BLN6321 showing fluorescence; (**B**), microscopy of brown rice 4233 no fluorescence. Va indicates the position of the vacuole lumen, while N indicates the position of the nucleus in cells.

The bright-field and confocal microscopy of the unstained cells isolated from a black cultivar **18BLN6321** revealed the fluorescence fully distributed in cells mainly inside the vacuole (Va), showing magenta color (Figure 4A), which tend to be darker where the concentration seemed to be high. To determine whether the presence of the fluorescence was due to anthocyanin, we performed the same experiment with brown rice **4233** with white stigma grown in the same condition. The results showed no fluorescence (Figure 4B). These were repeated three times at one-week intervals with different plants’ stigma, and the results were the same. Moreover, when the excitation was changed from 552 nm to 488 nm helium–neon and emission more than 700 nm, no fluorescence was observed. This observation showed that the coloration was essentially the result of the accumulation of anthocyanin in the cells, mainly large vacuole, which tended to occupy most of the cell’s volume.

### 2.4. Vesicular Structures Accumulating Anthocyanin in Stigma’s Cells

**Figure 5:**
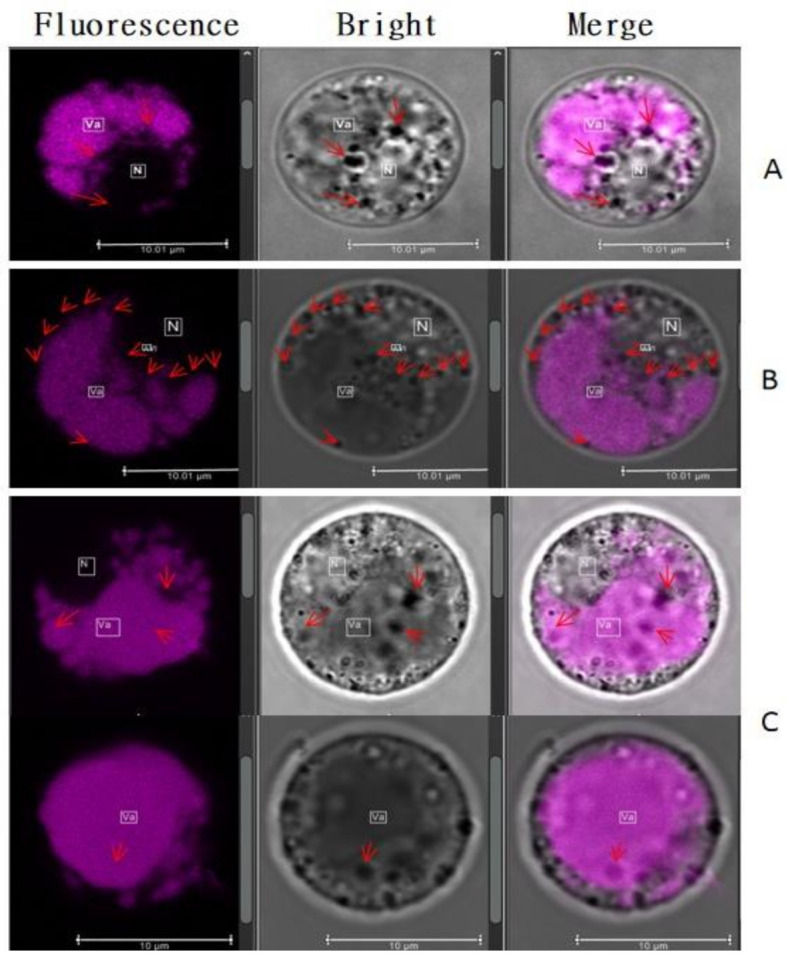
Distribution of anthocyanin structures inside the cells; the arrows indicate either anthocyanin prevacuolar vesicles (APV) or anthocyanic vacuolar inclusion (AVI); Va and N indicate the position of central vacuole and nucleus, respectively; (**A**), subcellular localization of soluble anthocyanin and body structures in the cell, red arrows here indicate the APV; (**B**), localization of APV alongside the tonoplast indicated with red arrows; (**C**), AVI in the diffuse anthocyanin, indicated with red arrows.

Autofluorescence of anthocyanin revealed two anthocyanin pools in the cells, the diffuse or soluble pools and the anthocyanin-filled body structures (Figure 5A). The distribution pattern of these pools of anthocyanin showed that the diffuse pools were observed only inside the vacuole lumen, and for body structure, we could identify a large number of anthocyanin-filled body structures in the cytoplasm, mostly alongside close to the tonoplast and few numbers inside the vacuole (Figure 5B). These were called anthocyanin prevacuolar vesicles (APV) and anthocyanin vacuolar inclusion to identify body structures inside the cytoplasm and the central vacuole, respectively. The estimated anthocyanin pools in different cells showed that anthocyanins were found mostly diffuse in the vacuole and engulfed in the APV inside the cytoplasm (Table 2). Our result remarkably showed very little AVI amounts in some cells and no AVI in other cells (Figure 5C), suggesting that most of the APV formed in the cytoplasm discard their content inside the vacuole, while other merges to the tonoplast and actively pass through different mechanisms. This result is completely in accord with the previous authors, who reported that cytoplasm is the synthesis site of anthocyanin, whereas vacuole is the accumulation site. Further characterization of APV and AVI highlighted that the color was very deep compared to diffuse anthocyanin inside the vacuole. This result suggested a high concentration and a different chemical component in vesicular anthocyanin compared to diffuse form, free inside the vacuole.

**Figure 6:**
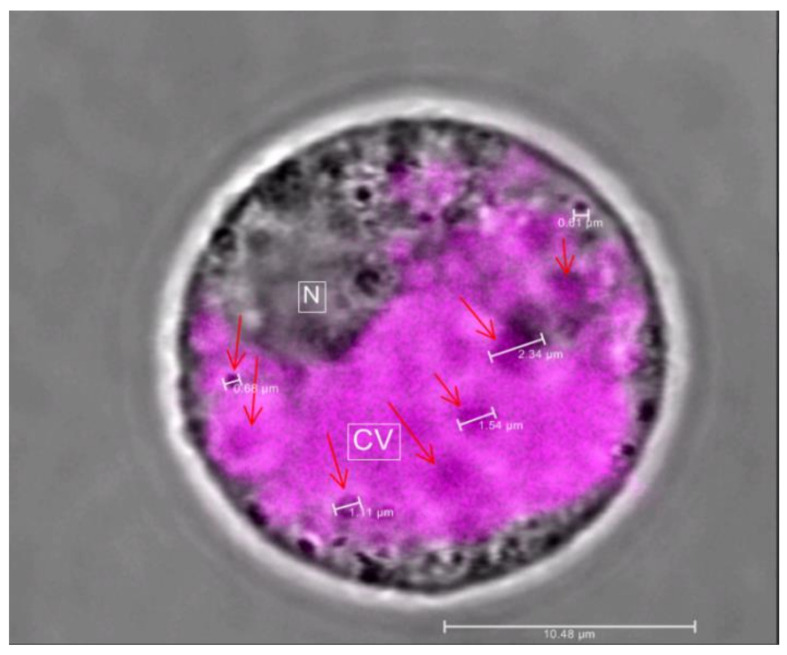
Identification and estimation of AVI sizes; CV and N are the position of central vacuole and nucleus, respectively; red arrows indicate different sizes of AVI identified.

Previous studies reported different shapes of AVI within the cell of the pigmented plants [22,23,24]. In this study, we investigated the form variation of anthocyanic structures in stigma cells of black rice. We have distinguished several structures with different spherical and irregular shapes having varying sizes, with the color tending to be more intense. The irregular shapes were dominant over the spherical shapes. Most irregular shapes were larger, while spherical shapes were very small. The thread-like structures were observed, but not in a significant amount. Most of the cells (35%) during our investigation had both diffuse anthocyanin (DA) in the vacuolar lumen and APV, while about 28% of cells had both diffuse anthocyanin, APV, and AVI (Table 2). The AVI sizes varied widely and were classified into three categories based on their diameter (Figure 6), namely, small size (S) with a diameter less than 0.5 μm, a middle size (M) with a diameter comprised between 0.5 and 1 μm and large size (L) more than 1 μm. Medium size and large size were predominant and found in both the vacuole and the cytoplasm.

## 3. Discussion

Anthocyanins are pigments considered as a model for genetics, molecular biology, and cell biology [37] to understand intracellular mechanisms associated with the trafficking of phytochemicals and serve multiple eco-physiological functions, including plant protection against abiotic stress [38] and visual signal for pollinators [18]. In this study, we took advantage of the unique autofluorescence properties of anthocyanin to investigate the subcellular localization of anthocyanin in pigmented rice through the stigma’s protoplast. Wide-field microscopy and confocal microscopy observations allowed the visualization of anthocyanin evenly distributed inside the vacuole lumen and cytoplasm. Stigma showed similar but not identical patterns of anthocyanin distribution inside the cells that have been previously reported in different model plant species [23,24,33], indicating that stigma’s protoplast accumulating anthocyanins is an appropriate model to study anthocyanin trafficking mechanisms in rice. Our results showed a spatial difference in the accumulation of anthocyanin. A high fluorescent pattern observed in the central vacuole, compared to the cytoplasm, suggested that the anthocyanin concentration in the main vacuole is higher than that in the cytoplasm. Several studies have highlighted that anthocyanin is synthesized in the cytoplasmic surface of the endoplasmic reticulum (ER) and stored in the vacuole lumen [18,28,39]. Our results corroborated with these studies indicating that vacuole is the site of accumulation. Like most flavonoid compounds, the trafficking and storage mechanisms of anthocyanin inside the vacuole lumen are similar to xenobiotic transport [40]. This mechanism ensures the detoxification of cells and avoids oxidation [41], which may be harmful to the cells, hindering the efficiency of their production [42], and enhancing the anthocyanin to function as pigments when present in the acidic vacuolar [43].

In some plant species, it was found that cells isolated from plant tissues containing anthocyanin exhibited subcellular structures in which anthocyanins accumulated discretely. These structures have received a variant name, called “anthocyanoplasts” for the first time [44], and are described as spherical intra-vacuolar membrane-bounds structures. Furthermore, it has been termed AVI [21]. In rice stigma, anthocyanin was found inside the cells in two forms: diffuse or free form and body or vesicular structures. We localized diffuse anthocyanin only in the vacuole lumen and not in the cytoplasm. It is believed that anthocyanin is aggregated and forms a body structure in the cytoplasm. A previous study suggested that the anthocyanins may be first packed into the prevacuolar compartment (PVCs) close to the anthocyanin biosynthesis sites in the cytoplasm [23]. They are also sequestered to the endoplasmic reticulum and endoplasmic reticulum-derived vesicle-like structures [24]. Thus, it is unlikely to find free anthocyanin inside the cytoplasm. Our results are in line with the finding of Markham his coworkers, who identified both soluble anthocyanin and vesicle-like structures of anthocyanin and reportedly, in most plants, these pigments are usually found dissolved uniformly in the vacuolar solution [21].

The feature of anthocyanin body structure has been investigated in some plant species. From more detailed examination in rice stigma, anthocyanins were observed mainly in two types of structures, differing mainly by their shapes, sizes, and localization in the cells; the irregular shapes that were identified mainly in the cytoplasm, while the spherical shapes were found both inside the vacuole and cytoplasm, and appeared smaller in size than irregular ones. It seems that irregular shapes are formed by the fusion of small body structures. This result conciliated with different authors. Therefore, variant names have been used to express these structures. Zhang and colleagues (2006) reported that these body structures were found in lisianthus in three forms: vesicle-like forms, irregular forms, and rod-like forms [23]. Compared to our observation in rice, we were not able to identify the rod-like form. Unlike Zhang and colleagues, who identified the body structures forming outside of the vacuole in the lisianthus flower as PVC, anthocyanin prevacuolar vesicle was considered in our study. When anthocyanins are formed on the ER, they seem to be concomitantly transported through the APV in the cytoplasm and stored as electron-dense vesicle-like bodies. This may explain why anthocyanin’s concentration is very high inside the vacuole that appears colorful compared to the cytoplasm. Gomez and colleagues (2011) highlighted that in grapevine, anthocyanins are accumulated in three types of vesicles localized inside and outside the vacuole called AVIs and APVs, respectively [45]. It was speculated that the APV moves actively in the cytoplasm and surrounding the vacuole alongside the tonoplast. This corroborated with our observations in which AVPs were mostly closer to the tonoplast. Only spherical forms called vesicle-like structures in suspension cells of sweet potato [25] and grapevine [45] have been deciphered with different sizes pooled in two categories GI and GII. Similar to Gomez et al. [45], our study revealed a spherical form that we categorized into three groups, namely, small, middle, and large sizes. Small sizes were mainly found outside in the cytoplasm in large quantities. This result is similar to the previously mentioned study where the GI (small size) was found mainly outside of the vacuole and thought to fuse each other to form the GII (large size). Other studies in *Arabidopsis* and maize revealed that cytoplasmic vesicles were also found in cells accumulating a high amount of anthocyanin [24,46,47]. Taken together, it can be concluded that anthocyanin can be sequestrated inside the cytoplasm and vacuole through the vesicle-like structures that differed from their shapes and sizes.

AVI’s external structure continuously fuels the debate and some contradictory microscopic observations about the presence of membrane [23,24,28,44,45,48] or absence of membranes [21,25] surrounding the anthocyanin-filled vesicles, which have still not received a clear elucidation. In the present study, our observations showed that the AVIs were remarkably distinguishable with deep color compared to soluble anthocyanin in the vacuolar lumen, implying that anthocyanin contained in AVI is a unique structure that neither can be merged or dissolved inside the vacuole. Moreover, the coloration of anthocyanic vesicular structures was different from that of vacuolar lumen or cytoplasm, suggesting a membrane encompassing the AVI that prevents dissolution.

The coloration of anthocyanin present in APV and AVI is denser than the diffuse anthocyanin. This may be due to the high concentration of anthocyanin inside the AVI and APV, as reported previously in similar studies, that the concentration of anthocyanin in AVI is very high compared to the vacuolar lumen [21,23,24,28,45]. Moreover, the differential pH levels between AVI and vacuole may also affect the color and intensity. Recent studies indicated that the in vivo coloration of anthocyanin was significantly affected by the environment’s pH [49,50,51,52], which in turn affected the fluorescence decay [33]. The fluorescence characteristics and properties depend on anthocyanin’s structure, which should be due to the flavylium cation and quinoidal bases species. The different forms present at higher pH values (mainly anionic charged forms) have the most interesting fluorescence properties [53]. Anthocyanins were red-pink in an acidic environment and blue in alkaline [37] because the flavylium cation becomes deprotonated as pH increase. Cruz et al., 2020 reported the color of C3G in the water at different pH, and the presence of PEGylated phospholipid micelles influences the stabilization and intensification effect of cyanidin 3-glucoside [54]. In plant cells, the acidity of the cytoplasm has been estimated to be approximately pH 7.1–7.8, and that of the vacuole approximately pH 4.9–6.0 [55,56,57,58,59]. Recently, Wahyuningsih et al., 2017 highlighted the effect of pH on the coloration of anthocyanin. According to that study, anthocyanins are red for pH < 3, magenta when pH is comprised between 3 to 6, violet or purple in neutral solution, and blue in alkaline pH [60]. In our study, the fluorescence varied from magenta to purple and C3G was the predominant anthocyanin. Conclusively, we inferred that the difference in coloration between the diffuse anthocyanin and AVI observed in our results was due to the pH. Since the membranous AVIs are formed in the cytoplasm, the vacuole’s acidity may not affect its coloration and intensity. Pigmentation and fluorescence of free anthocyanins are affected once they are deposited inside the vacuolar lumen. This may explain why AVI and APV were deeper than diffuse anthocyanin.

The coexistence of free anthocyanins and AVI inside the vacuole may be due to the presence of two transport mechanisms from the cytoplasm to the central vacuole. On one side, anthocyanins may be deposited as vesicle-like body structures called AVI. In contrast, on another side, the release of pigment may occur after the rupture of the structure encompassing the body structure followed by merging with the vacuolar solution that becomes part of diffuse anthocyanin. Recently, Grotewold and Davies suggested ligandin transportation and vesicular transportation as two ways by which anthocyanin are delivered to the central vacuole [44]. The first transport mechanism involves ligands that escort anthocyanin products to the vacuole and sequestrate into AVIs, mostly when anthocyanin concentration is too high. It was speculated that anthocyanin first bounds to suitable GST and moves through active transport until it reaches the tonoplast. The second mechanism involves vesicles that drop their cargo transported to the vacuole [44]. Several studies supported that AVI in vacuole occurs as an autophagy mechanism of intact vesicles called microautophagy as the classical autophagic mechanism is unlikely to be involved in the formation of AVIs [23,24,28]. In this process, APV in close contact with the vacuolar surface is directly engulfed by the vacuolar membrane in a process reminiscent of microautophagy. During the process, the APV is surrounded by a single membrane, which is derived from the tonoplast and ultimately becomes free in the vacuolar lumen like an autophagic body [28]. However, it has been elucidated that the autophagic process impeded the formation of AVI [27]. Another study highlighted that the formed autophagic bodies are broken down by vacuolar resident hydrolases [61], causing the release of anthocyanins into the vacuolar sap. Thus, as it was reported for the case of yeast aminopeptidase I (AP.I) resident vacuolar enzyme [62], we deductively can conclude that the phenomenon of the cytoplasm-to-vacuole autophagic mechanism may be involved in the import of hydrolytic enzymes contributing to autophagic body breakdown leading to diffuse anthocyanin inside the vacuole lumen observed in this study. It seems like during the process, the types of anthocyanin transported would influence the fate of the aggregates. Unstable anthocyanin may easily be broken-down, while stable anthocyanin remains bind inside the vacuole. In this study, anthocyanin quantification in stigmas revealed that C3G was dominant with a higher concentration than P3G. C3G, which is hydroxylated anthocyanin, is unstable than P3G, which is a methylated one. As reported previously, methylation of the phenolic B ring enhances stability, reduces reactivity, increases water solubility, and subsequently reinforces its color properties [63,64]. Glycosylation affects stability through the number and position of sugar moieties on the molecule. Hence, diglucoside at C3 is more stable and stronger than monoglucoside, while C5 decreases pigment intensity [65]. This may explain why anthocyanin was mostly found diffuse inside the vacuole. Other studies reported that AVIs serve a specific role in aggregating or sequestrating anthocyanins with particular modifications and selectively accumulated some distinct type of anthocyanins, such as acylated anthocyanin identified in grapevine suspension cultures [26] and C3G and its derivatives [27] shown to strongly correlate with the formation of AVI. C3G was the dominant anthocyanin, but fewer AVIs were found, suggesting no direct relationship between C3G and AVI, as reported by previous authors. Considering that the trafficking mechanism of anthocyanin from the cytoplasm to the central vacuole is mostly by autophagy, they may be as much as anthocyanin in the cytoplasm compared to the vacuole. Our result is contrary to the previous statement suggesting different mechanisms of anthocyanin trafficking to the vacuole. The first one only sequestrates some particular anthocyanin and store as AVI [26,45], and the other one release anthocyanin in the central vacuole in the diffuse form [27]. The few numbers of AVI in the vacuole compared to the number of APV in the cytoplasm and a large amount of diffuse anthocyanin uniformly distributed in vacuole hypothesized that during the passage of APV from the cytoplasm to the vacuolar lumen, some vesicular structures might rupture, and anthocyanins that are released, subsequently dissolve inside the vacuole lumen.

## 4. Materials and Methods

### 4.1. Plant Material

In this study, two different rice (*Oryza sativa* L.) cultivars were used, namely a black rice “18BLN6321” with deep purple stigma and black caryopsis and a brown rice 4233 with white stigma and brown caryopsis. Rice plants were cultivated in the experimental field at Guangxi University, Nanning city, Guangxi province (latitude: 22°49′0.01″ N, longitude: 108°19′0.01″ E), China. This site encompasses a warm, monsoon-influenced humid semitropical climate with an annual average temperature of 21.83 °C (71.3 °F) with an average annual precipitation of 1190 mm. Rice plants were grown in the paddy field from August to November 2020. At the flowering stage, entire plants from both cultivars were removed from the soil and immediately transferred in the pot with soil and water, then carried to the laboratory where some traits, such as leaves, stems, caryopsis, and flowers colors, were investigated. Afterward, stigmas were collected for anthocyanin quantification and protoplast extraction.

### 4.2. Method

#### 4.2.1. Characterization of the Pigmented Black Rice Tissues

We investigated the anthocyanin pigmentation in rice leaf sheath, leaf blade, ligule, stem, stigma, and caryopsis. Frozen section or cryo-section method with cryostat microtome was used for sample preparation. Briefly, plant material was collected from the field and cut to an appropriate length and then transferred into a 10% sucrose solution with 0.3% Tween-20 (PBS). Samples (1–3 cm) were cut and embedded into the optimum cutting temperature (OCT) compound on the stage of cryomold inside the cryo-microtome. Additional OCT was poured onto the sample on the cryo-microtome stage (−20) and remained until it gets completely frozen. The frozen samples were mounted on the cryo-microtome and trimmed with a disposable tungsten carbide blade (Jung TC-65, Leica Instruments) until the desired depth. The cut section (10–50 μm) was then transferred on a glass slide in the cryo-chamber. Sections were observed under super depth three-dimensional (3D) microscopic imaging system and processed further using Adobe Photoshop 2020 (Adobe, http://www.adobe.com, accessed on 18 February 2021).

#### 4.2.2. Anthocyanin Quantification in Rice Stigma and Caryopsis

The anthocyanins were quantified in the stigma and caryopsis using high-performance liquid chromatography method, and three types of anthocyanin glucoside standard, namely, cyanidin-3-O-glucoside chloride, peonidin-3-O-glucoside chloride, and petunidin-3-O-glucoside chloride, were used. Briefly, 1 g of sample was pretreated by adding 50% ethanol–water (containing 1% formic acid), then mixed using a vortex mixer for 30 s, ultrasonic for 30 min. Samples were centrifuged at 13,000× *g* rpm for 30 min; the supernatants were combined and filtered using a 0.45 mm nylon filter. A 30 μL aliquot of samples was subsequently injected through the membrane of the HPLC system. The compound separations were achieved on 250 mm × 4.6 mm i.d., 5 μm reversed-phase Waters XBridge C18 with 1% formic acid and acetonitrile used as mobile phase. Elution was processed with solvent A, 1% formic acid–water, solvent B, 1% formic acid–acetonitrile. The gradient profile for the separation of anthocyanin was 92% A–8% B (0 min). The gradient profile was subsequently changed linearly to 20% A–80% B for 20 min, maintained for 2 min, and returned to 92% A–8% B, which was maintained for 8 min. The column temperature was kept at 35 °C; the flow rate was 0.8 mL · min^−1,^ and chromatograms were recorded at 530 nm. Anthocyanins were quantified by comparing the chromatograms to external standards, and the experiment was repeated three times.

#### 4.2.3. Protoplast Isolation

Stigmas protoplast were isolated following Yoshida et al. [49] and Poutska et al. [24] with slight modification. Rice stigmas were collected and transferred in a 2 mL centrifugation tube containing 1.5 mL enzymatic solution (0.6 M mannitol, 10 mM MES, cellulase RS 1.5%, macerozyme 0.75%, 0.1% BSA, and 1 mM CaCl_2_, pH 5.7) and kept in the incubator for 6 h at 28 °C with 40–50 rpm shaking. After digestion, 1.5 mL of W5 solution (154 mM NaCl, 125 mM CaCl2, 5 mM KCl, 2 mM MES) was added, gently mixed, and filtered into new Petri dishes using 150 mesh sieve (molecular sieve). The filtrate was transferred to a 10 mL round-bottomed centrifuge tube, and protoplasts were centrifuged at 400 rpm for 5 min at 25 °C. Extra W5 was completely collected and poured out, then a small amount of new W5 was added to the pellet at the bottom of the tube, and the sample was mixed well.

#### 4.2.4. Microscope Observation

Leaves, stems, ligule, and caryopsis were investigated after longitudinal and cross-section (10–50 μm) using cryo-microtome, then unstained sections were imaging through a super depth three dimensional (3D) microscopic system. The cellular and subcellular localization of anthocyanin were evaluated using a confocal laser scanning microscope (LEICA-TCS-SP8MP, Wetzlar, Germany) with a Leica HyD detector. All samples were mounted on glass slides and covered with glass cover slides for observation. Excitation was provided by a helium–neon 552-nm laser and emission long pass 610–670 nm laser for the autofluorescence of anthocyanin magenta to purple autofluorescence obtained from cells. The sections were observed with plan motCORR a Leica 40x/0.85 objectives. Pictures were scanned (512 × 512 pixels), saved, and processed using the Leica Application Suite X software version 3.7.2 build 22383 (https://webshare.leica-microsystems.com/latest/core/widefield/ accessed on 18 February 2021). About 150 cells were observed, and the experiment was repeated three (3) times.

## 5. Conclusions

Using nondestructive cell isolation and modern microscopy techniques, we examined the rice stigma’s protoplast containing anthocyanins and obtained images of anthocyanin distribution inside the cells. Although the concentration of C3G is higher in the stigma than the caryopsis, the type of anthocyanin in caryopsis and stigma was the same. Suggesting that stigma cell is an excellent system for in vivo studying anthocyanin in rice caryopsis. Anthocyanin is packed in the cytoplasm in the form of APV in proximity to the tonoplast. In the main vacuole, AVI and diffuse anthocyanin are found evenly distributed. AVI results in a deeper hue color than diffuse anthocyanin, which may be related to the concentration and pH difference between both forms of anthocyanin in the vacuolar lumen. Together, these results were evidence of the existence of different transport mechanisms involving several transporters. Our results provide a good foundation to understand anthocyanin metabolism in plants, sequestration, and trafficking in black rice. To clarify these mechanisms in black rice cells, further investigation is currently being undertaken to look into different transporters involved in this process.

## Figures and Tables

**Table 1:** Anthocyanin content (µg. g ^−1^ FW) in purple stigma, black, and white caryopsis.

Type of Anthocyanin	Purple Stigma	Mature Black Caryopsis	Mature White Caryopsis
Pt3G	ND	ND	ND
P3G	35 ± 3	35.98 ± 7	ND
C3G	16,929.14 ± 35	814.59 ± 5.5	ND

Anthocyanin content is represented as the value ± SD of three biological replicates; ND, not detected; Pt3G, petunidin-3-O-glucoside; P3G, peonidin-3-O-glucoside; C3G, cyanidin-3-O-glucoside.

**Table 2:** Observation of different pools of anthocyanin in cells.

Compartments	Number of Cells Observed			Total Cells	Percentage of Cells
	N1	N2	N3		
DA	15	11	9	35	23%
DA + APV	25	15	12	52	35%
DA + AVI	7	4	0	11	7%
DA + APV + AVI	23	11	8	42	28%
AVI	0	0	0	0	0%
APV	6	4	0	10	7%
APV + AVI	0	0	0	0	0%
Total	76	45	29	150	

Cells were sorted and counted base on the presence of different forms of anthocyanin inside the cytoplasm and central vacuole; N refers to the number of observations; Da means diffuse anthocyanin inside the central vacuole; AVI, anthocyanin vacuolar inclusion; APV, anthocyanin prevacuolar vesicle.

## Data Availability

Not applicable.

## Abbreviations

AP.I	Aminopeptidase I
AVI	Anthocyanic vacuolar inclusion
C3G	Cyanidin-3-O-glucoside
ER	Endoplasmic reticulum
FLIM	Fluorescence lifetime imaging microscope
FW	Fresh weight
GST	Glutathione-S-transferase
HPLC	High-performance liquid chromatography
LT	Ligandin transportations
MATE	Multidrug and toxic compound extrusion
MES	N-morpholino-ethanesulfonic acid
OCT	Optimum cutting temperature
P3G	Peonidin-3-O-glucoside
PBS	Phosphate-buffered saline
pH	Potential of Hydrogen
PVC	Pre-vacuolar compartment
ROS	Reactive oxygen species
TAC	Total anthocyanin content
VT	Vesicular transport
